# The first pediatric emergency and critical care fellowship in sub-Saharan Africa: investing in local leaders to build subspecialty capacity in low- and middle-income countries

**DOI:** 10.3389/fped.2026.1847261

**Published:** 2026-06-03

**Authors:** Bhupi Reel, Rashmi Kumar, Mardi Steere, Emily A. Hartford, Arianna Shirk, Amelie von Saint Andre-von Arnim

**Affiliations:** 1 Department of Paediatrics and Child Health, University of Nairobi, Nairobi, Kenya; 2 Paediatric Emergency Department, Women’s and Children’s Hospital, Adelaide, SA, Australia; 3 Charles Darwin University, Menzies School of Medicine, Darwin, NT, Australia; 4 Department of Pediatrics, Division of Emergency Medicine and University of Washington, Seattle Children’s Hospital, Seattle, WA, United States; 5 Department of Pediatrics, AIC Kijabe Hospital, Kijabe, Kenya; 6 Department of Pediatrics, Division of Critical Care Medicine and University of Washington, Seattle Children’s Hospital, Seattle, WA, United States

**Keywords:** capacity building, global healh, low and middle income countries, low resource settings, medical education, pediatric critical care, pediatric emergency medicine, sub Saharan Africa

## Abstract

Nearly 7 million children die annually worldwide mostly in the global South predominantly from treatable conditions. Pediatric emergency and critical care (PECC) systems are critically underdeveloped across low- and middle-income countries (LMICs), where healthcare worker shortages, limited infrastructure, and delayed presentation contribute to high rates of early in-hospital deaths. Investment in locally trained PECC physician leaders is essential to building sustainable subspecialty capacity in the region. In 2019, the University of Nairobi (UoN), in partnership with Kenyatta National Hospital (KNH), AIC Kijabe Hospital (AIC KH), the University of Washington (UW), and Seattle Children's Hospital (SCH), established the first PECC fellowship program in sub-Saharan Africa (SSA). Curriculum development followed a modified Kern framework, contextualized for the East African setting. The two-year program integrates core clinical rotations, online asynchronous learning modules, virtual lectures from global faculty, quarterly in-person simulation-based intensive weeks, and training in research, quality improvement, and leadership. To date, 14 fellows from 5 countries have graduated. All graduates are practicing in SSA; 82% work in public facilities, and 64% are the only PECC-trained physicians at their institution. Fellow satisfaction surveys indicate strong ratings for clinical exposure, supervision, and leadership training. Both training sites have expanded their critical care infrastructure. The PECC-Kenya fellowship demonstrates that rigorous, locally anchored subspecialty training in PECC is achievable in a resource-limited setting through sustained global partnership. This model may serve as a replicable blueprint for building pediatric emergency and critical care capacity across SSA and other LMICs.

## 1 Introduction

Globally, nearly 7 million children and youth die each year; with approximately 80% of these deaths concentrated in sub-Saharan Africa (SSA) and Southern Asia (1). In low- and middle-income countries (LMICs), most child mortality is attributable to treatable conditions including respiratory infections, malaria, diarrhea, sepsis, injuries, and neonatal complications (1, 2). Pediatric emergency and critical care (PECC) involves the management of life-threatening conditions, yet healthcare systems across LMICs frequently lack the resources, clinician expertise, and infrastructure to effectively care for critically ill children. Compounding this challenge, limited access to care often results in delayed presentation, with children arriving at health facilities in advanced, life-threatening stages of illness. Consequently, up to 80% of in-hospital deaths occur within the first 48 h of admission (3–6).

The World Health Organization (WHO) has recently undertaken strategic initiatives to prioritize emergency and critical care development for health systems (7, 8) with the introduction of the Basic Emergency Course, the Emergency Care Toolkits and the Acute Care Action Network (ACAN) (9). Published outcomes from short courses in emergency and critical care include positive educational impact, feasibility, and adaptability; while sustained impact is harder to achieve (10–12, 27). There is an urgent need to invest in the education and retention of local specialty trained PECC physicians to lead the introduction and scaling of crucial healthcare services in essential emergency and critical care across LMICs (13, 14). This need is particularly acute in SSA, where severe healthcare worker shortages persist — with only 0.23 physicians and 1.15 nurses per 1,000 people, compared to 3.58 and 9.81, respectively, in high-income country (HIC) settings (15).

General pediatrics training in SSA usually takes 3–4 years following medical school and internship. Pediatric subspecialty fellowship training is still new in the region; most programs have a two-year duration. Expanding subspecialty medical education for PECC physician leaders is critical in a region where most countries have few or no such trained specialists (3–5). Until recently, South Africa was the only country in SSA offering fellowship-level training in pediatric critical care, and no pediatric emergency medicine training programs existed anywhere on the African continent (16–18).

In 2019, the University of Nairobi (UoN) in collaboration with Kenyatta National Hospital (KNH), AIC Kijabe Hospital (AIC KH), the University of Washington (UW), and Seattle Children's Hospital (SCH), established a novel two-year fellowship training program in PECC. This perspective describes the development and implementation of this first-of-its-kind program in SSA.

## 2 Methods

### 2.1 Training sites

KNH is the largest government referral hospital in Kenya and the main training site for the PECC fellows, who are enrolled at the UoN. KNH has approximately 15,000 general pediatrics 500 PICU admissions annually. The latter is limited by a 5 bed PICU. PECCfellows and faculty also co-manage critically ill children in the cardiac, surgical and adult ICUs, as well as in the acute rooms on the pediatric wards. Fellows manage critically ill patients as they present in the emergency unit and through their ICU admission. KNH has multiple pediatric subspecialty services where fellows can also rotate. At the time of PECC program development, the only other existing fellowship program at UoN/KNH was in pediatric anesthesia.

AIC KH is a 296-bed non-profit hospital system in rural Kenya with a large range of medical and surgical services and educational programs. At AIC KH, fellows manage emergently ill children as they present in casualty and during their admission in the ten-bed pediatric intensive care and high dependency unit (HDU). AIC KH admits about 1000 pediatric patients annually, with 100 requiring PICU and 400 HDU care.

### 2.2 Curriculum development

We used a modified stepwise Kern approach for medical educational curriculum development to provide a structured framework for this novel fellowship. Faculty members from various subspecialty backgrounds, institutions and nationalities convened at UoN in a series of workshops to collaboratively develop a PECC curriculum plan. Curricular elements from international certification organizations in PECC were used as a benchmark and adapted for local context. Additional steps were incorporated to address global partnership formation and program funding, as detailed below:

1. *Defining the need and scope for the PECC fellowship program* at UoN
2. *Establishment of the PECC Kenya global partnership*
3. *Formulation of goals and objectives*
4. *Development of educational structures*
5. *Securing funding*
6. *Program implementation*
7. *Program evaluation and monitoring*

### 2.3 Faculty oversight

The PECC Kenya fellows were initially supervised by three local faculty, two PICU physicians at UoN/KNH and one PEM physician based at AIC KH. In 2022 and 2025, two fellowship graduates joined the faculty at KNH. Faculty oversight from UW includes the co-founding PICU physician and a lead physician in PEM. A part-time program coordinator based at SCH manages collaborative meetings, lecture schedules, visiting trainers and fellows, and budgets.

### 2.4 Fellow and program evaluation

Fellows take written exams and simulated patient clinical case exams after year 1 and 2, evaluated by program faculty. Individual rotations are graded on a pass/fail basis with feedback given by supervising local faculty. Fellows complete an anonymous biannual Likert scale-based survey (see supplement) to assess the program's effectiveness (Seattle Children's IRB ID: STUDY00005897).

## 3 Results

### 3.1 Defining the need and scope for the PECC fellowship program

There is an extremely high volume of critically ill pediatric patients at KNH. The introduction of the first specialist in critical care in 2012 brought significant improvements in survival for children admitted in the adult ICU (mortality decline from 61.8 to 23%) and later in the dedicated pediatric ICU (mortality decline 76.2 to 37.5%) (19). These substantial reductions in mortality provided evidence for the urgent need for a regional PECC fellowship program. From 2012 to 2013 founding faculty members performed informal needs assessments through conversations with consultants, physician trainees and nurses at urban, rural, private and public, sub-county, county and referral hospitals in Kenya to identify educational needs, opportunities and challenges in the design and implementation of a PECC curriculum. The main finding was that the limited expertise, leadership and systems for recognizing and managing critically ill children across health facilities in Kenya was leading to delays in care, contributing to the high morbidity and mortality in this patient population. Based on this assessment, the team decided to integrate pediatric emergency medicine with critical care to provide a continuum of care for critically ill children.

### 3.2 Establishment of the PECC global partnership

The UoN and UW have had long standing partnerships in research and education with a Memorandum of Understanding (MOU) already in place. It was through this history of institutional collaboration that Kenyan faculty requested support for this novel training program. The PECC Kenya partnership between UoN and UW/SCH then developed with the aim of improving outcomes for critically ill and injured children in sub-Saharan Africa. AIC KH was integrated into the partnership as a site for fellows to experience working in a different hospital system and because it had the only physician trained in pediatric emergency medicine at the time in Kenya. Subsequently, in 2013 an MoU was signed between UoN and AIC Kijabe Hospital.

### 3.3 Formulation of goals & objectives

The vision and mission of the PECC fellowship were developed through multiple collaborative meetings from 2013 to 2015. The vision of the PECC-Kenya partnership was to improve the outcomes of critically ill and injured children in sub-Saharan Africa by fostering advancements in education, research, advocacy, and clinical service. The partnership's mission was to ensure the delivery of optimal care for critically ill children across health facilities in the region by increasing PECC capacity through the PECC fellowship at UoN in Kenya.

In the short term (5 years), the fellowship aimed to increase the number of PECC doctors in Kenya and lay the groundwork for becoming self-sustainable. Medium term objectives (5–10 years) included the reduction of child mortality rates at PECC training sites and the implementation of institutional protocols and infrastructure for critically ill children. Long term goals (10 years and beyond) included an increase in the number of PECC specialists across SSA with creation of a network to decrease child mortality through shared educational resources, collaborative research, and expansion of regional PECC infrastructure.

### 3.4 Development of educational structures

A curriculum development working group included local and international specialists in pediatric anesthesia, critical care, emergency medicine, pulmonary medicine, cardiology and endocrinology, representing multiple institutions including UoN, KNH, AIC KH, the Kenyan Ministry of Health, the Kenya Paediatric Association, UW and SCH. The group used established critical care and emergency medicine curricula from HICs as an initial guide for curriculum development. Subgroups rigorously reviewed each topic, assessed relevance for the East African context, then discussed with the entire curriculum workgroup until consensus was reached on inclusion, exclusion or modification. The group added locally relevant topics based on disease spectrum and available resources and created a fellow rotation schedule to include clinical rotations at KNH and AIC KH, an international elective in a higher resource setting, and blocks for leadership, implementation science and quality improvement training (Table 1). The 2-year curriculum was further revised to match national guidelines based on a detailed multi-year review process by the UoN's Department of Paediatrics and Child Health, the curriculum team, the School of Medicine Board, and UoN Senate. In 2018, the curriculum was approved, allowing for trainees to receive accreditation in PECC from UoN upon completion of the fellowship. Throughout the approval process, PECC faculty worked to recruit volunteer trainers, to produce self-guided, online asynchronous PECC learning modules, and design in-person intensive training weeks to commence upon approval of the fellowship.

**Table 1:** PECC-Kenya rotation schedule over 2 years.

ROTATION	DURATION (MONTHS)
Pediatric ICU with PEM consultation, Kenyatta National Hospital	10
Emergency care, PICU/HDU, AIC Kijabe Hospital	3
Private hospital PICU	1
Anaesthesia	1
Renal	1
Leadership and research	1
Cardiac critical care mission	1
Cardiology	1
Transport medicine	1
Electives (including international rotation)	2
PEM intensive weeks (8 × 1 week)	2
Total	24

PECC, pediatric emergency and critical care; PEM, pediatric emergency medicine; PICU, pediatric intensive care unit; HDU, high dependency unit.

### 3.5 Securing funding

Funding for medical education including trainee salaries, infrastructure development, curriculum enhancement, and faculty oversight is extremely limited across institutions in SSA (20). Postgraduate medical trainees in Kenya pay tuition and do not routinely receive stipends. Therefore, funding for a sustainable and rigorous program must include trainee support sufficient to eliminate the financial necessity of concurrent private sector employment. The initial start-up funds were obtained through seed funding from private foundations and event-based fundraising in Seattle. The UoN secured philanthropy funding for Kenyan fellow support which bonds them to working in regional government referral settings upon fellowship completion. Four fellows have been supported by their country governments or local institutions to return and build the specialty. The UW Department of Pediatrics has provided funding for UW faculty travel, program coordination, and one annual fellow stipend per year. Unfortunately, PECC-Kenya has not yet been able to secure enough funding to pay faculty program leadership at UoN or UW.

### 3.6 Program implementation

#### 3.6.1 Recruitment process

Fellowship applicants are required to hold a master's degree in pediatrics, which is a minimum of 3 years of post-internship training in pediatrics in an accredited University program, and at least one year of clinical work experience. Initial recruitment in 2018 occurred through personal and professional networks, social media, and national societies. Since then, we have received applications from pediatricians from across multiple countries in SSA. The selection process, managed by the UoN, includes an online application and remote interviews. Thus far, we have recruited 2–4 new fellows per year depending on demand, best match and stipend availability.

#### 3.6.2 Curriculum implementation

Curricular goals are met through core clinical care rotations at KNH and AIC KH with bedside teaching mostly by Kenya-based faculty (Table 1), through online self-guided modules, a live virtual lecture schedule, and quarterly didactic intensive weeks with in-person teaching and simulation. Self-guided online modules are hosted on an internal UW course website and cover curriculum topics with narrated slides, quizzes, and suggested reading. Virtual live lectures are held 2–3 days per week including curriculum topics, board review, journal clubs, and case presentations, given by over 100 volunteer lecturers from over 40 institutions globally. Monthly virtual simulation sessions using the virtual resuscitation room reinforce teamwork and resuscitation training (21). While the formal UoN curriculum has not been changed since its approval, the emphasis and mode of training have been adjusted over time based on available clinical resources such as point of care ultrasound, based on new learning opportunities through some of the local and visiting surgical teams, and based on fellows’ feedback leading to incorporation of regular virtual simulation and board review sessions. As of 2024 we have started collaborative implementation of virtual training resources with a new PECC fellowship at St. Paul's Millennium Hospital in Addis Ababa, Ethiopia.

Quarterly didactic intensive weeks, conducted at the AIC KH's high-fidelity simulation center focus on emergency medicine and hands-on training, led by local and visiting faculty. Fellows have no clinical duties during these weeks and undergo training in point of care ultrasound, airway management, and resuscitation. Participation in biannual cardiac surgical missions at MP Shah Hospital, a private institution in Nairobi, with a team of visiting cardiac surgeons, cardiologists, anesthetists and nurses through Healing Little Hearts introduces senior fellows to perioperative care of common congenital heart diseases (22).

Fellow rotations from Kenya to the UW, initially postponed due to pandemic travel restrictions, commenced in 2024 and aim to expose fellows to different healthcare systems. They shadow faculty in emergency departments and intensive care units, and participate in regular PEM/PCCM didactics, simulation, point of care ultrasound education. They gain exposure to operational aspects of the ED and ICU, medical education, family centered rounds, pre-hospital care and transport programs, and clinical support programs such as respiratory therapy and child life specialists.

To prepare fellows to drive change in their healthcare system, they undergo training in basic clinical research, quality improvement, implementation science and leadership skills, and complete a scholarly project.

#### 3.6.3 PECC fellows and graduates

From its inception, the PECC Kenya program aspired to be a regional center of excellence for training. Thus far, 12 fellows from 5 countries have graduated, 8 from Kenya, and one each from Rwanda, Tanzania, Ghana and Nigeria. Current fellows are from Kenya (5) and Sierra Leone (1). National medical licensing and immigration approvals are crucial to the success of a regional program; approval of non-Kenyan credentials goes through the Kenyan Commission for University Education (CUE) (23). All 12 PECC graduates work in SSA, 9 (82%) in public facilities, 7(64%) as the only PECC doctor at their facility (Table 2). Two graduates were hired by KNH and have become essential as faculty in the fellowship program. Other graduates are working on developing their own PECC or PEM training programs in Ghana, Rwanda and Tanzania. All non-Kenyan graduates are the first physicians with PECC training in their countries while all Kenyan graduates outside KNH are the only PECC trained doctors in their institutions.

**Table 2:** Characteristics and work engagement of PECC Kenya fellowship graduates.

Country of Origin *n* (%)	Public Hospital *n* (%)	Private Hospital *n* (%)	Tertiary Care *n* (%)	Academic Institution *n* (%)	PEM/PCCM Colleagues at Same Institution *n* (%)
Kenya 10 (71) Rwanda 1 (8) Tanzania 1 (8) Nigeria 1 (8) Ghana 1 (8)	11 (79)	3 (21)	7 (58)	6 (50)	5 (35)

PCCM, Pediatric Critical Care Medicine; PEM, Pediatric Emergency Medicine; PECC, Pediatric Emergency and Critical Care.

### 3.7 Program monitoring and evaluation

Biannual program evaluations are reviewed by the faculty oversight team and curriculum adjustments are made in response as able. Since launching PECC-Kenya, we have had a total of 33 survey responses from fellows, a response rate of 75%. Most responses rated the program as well organized for training [29 (87%)] and the clinical exposure adequate for learning [31(93%)]. Most [27(81%)] were satisfied with their level of clinical supervision. Sixty percent (20) rated the available hospital resources sufficient. The majority [28(84%)] reported learning important aspects of leadership and improving health care systems.

Fellows complete written exams developed by the program faculty at the midpoint and at the end of their fellowship. To assess clinical competencies, trainees complete annual graded simulation sessions on key pediatric cases in emergency and critical care. The UoN requires a passing grade above 50% and all fellows have thus far successfully completed their final written and practical exams. PICU patient outcomes are followed via an electronic database supported by our program at AIC KH, while at KNH major morbidity and mortality data is currently followed by their PICU staff over time with a more elaborate electronic database being planned. The PECC-Kenya program is keeping in close touch with all its graduates, with formal annual surveys on their PECC infrastructure and guideline developments, as well as institutional mortality trends in production to help monitor all program goals over time.

## 4 Discussion

PECC-Kenya is an international collaboration focused on training leaders in PECC in Sub-Saharan Africa. It successfully developed and implemented the first PECC training program on the African continent with 12 graduates currently working in 5 countries mostly in the public sector. The keys to the program's success include strong global partnerships; dedicated local, visiting, and remote faculty; a contextualized curriculum created by providers in resource constrained settings; hospital facilities for hands-on training; virtual and in-person learning opportunities and living stipends for fellows.

Despite the high burden of pediatric critical illness in SSA, there are few options for clinicians to pursue PECC specialty training on the continent. To the best of our knowledge this includes only three other programs: the well-established African Pediatric Fellowship Program at the University of Cape Town and Red Cross War Memorial Children's Hospital (24); and the PECC fellowship programs at St. Paul's Millennium Hospital in Addis Ababa, Ethiopia and the Ghana college of physicians and surgeons in Kumasi which both launched in 2024. We hope that the PECC-Kenya model may serve as a blueprint for further programs in the region with a goal for collaborative training opportunities amongst institutions and we encourage other sites to contact us.

Successful PECC systems require not only strong and well-trained physicians but also nurses, mid-level providers and an EMS infrastructure. Kenya is unique in the region as it offers pediatric critical care nursing and pediatric critical care and emergency clinical officer (mid-level provider) training, providing further depth, sustainability and cost-savings to the PECC infrastructure (25).

The PECC Kenya collaboration was founded with the idea of training experts on a continuum of emergency to critical care medicine to meet the needs in the region. Thus far, graduates have found this training helpful and applicable to their work as they create systems to enhance early recognition and intervention of pediatric critical illness upon presentation and improve the infrastructure to continue ongoing effective life-saving treatments. Emergency medicine is a new specialty in Kenya and thus far most of our graduates are based in an ICU while consulting or overseeing the delivery of emergency care. However, as the specialty grows, PECC Kenya fellowship graduates can further utilize their training in enhancing transport and disaster medicine, patient triage and flow, and procedural competencies.

After 6 years of operation, the PECC-Kenya fellowship has achieved its short-term goals of increasing the number of local PECC doctors and trainers in Kenya and moving towards program sustainability with a hybrid virtual/in-person training approach. The medium-term objectives of tracking and reducing child mortality rates at PECC training sites are underway with a patient outcome database fully functional at one of the two training sites. Both sites have expanded their critical care infrastructure in bed capacity and PECC clinical protocol implementation, and a 22-bed pediatric ICU is currently being constructed at KNH. These advances are only possible through advocacy of PECC faculty and graduates, an example of the important role ofclinical leaders in LMICs in key service delivery decisions of the complex problems they face (26). PECC graduates will be well positioned to lead the new WHO ACAN initiatives for their respective regions as well as the country specific implementation. We foresee a future network of PECC specialists across SSA to work together to improve infrastructure and outcomes and address crucial gaps in evidence-based management and guidelines of critically ill children in their region. Collaborative research projects between multiple PECC graduates’ clinical sites have been developed and submitted for funding. We are hopeful fellows, graduates, and additional institutions will continue to collaborate on research and educational initiatives that can increase local evidence, strengthen the regional PECC network, and ultimately improve outcomes for PECC in the region.

### 4.1 Limitations

While we have successfully trained PECC subspecialty providers, the PEM training portion is focused on management of severely ill patients as current institutional structures in Kenya offer a smaller range of interventions in the emergency department and PEM is only starting to develop as a subspecialty in SSA. Another limitation is the inherent complexity of measuring the patient impact of having physicians trained in PECC. We are still working on improving monitoring and evaluation systems to follow short- and long-term outcomes of graduates’ work. The duration and clinical demands of the fellowship have made it challenging for fellows to publish their research and quality improvement projects. Funding has been limited; we prioritize fellow living stipend support and educational materials followed by travel in both directions.

## 5 Conclusion

There is a huge need for capacity building in PECC in SSA to improve child survival from treatable conditions. Sustainable strengthening of critical care and emergency medicine is a gradual and resource intensive process. Collaborative global partnerships like PECC-Kenya can help implement pediatric subspecialty training in LMICs. The PECC-Kenya path and vision of improving care and outcomes of pediatric critical illness in SSA may serve as a blueprint for similar emerging programs with a goal of networking and sharing resources between sites and global regions.

## Data Availability

The raw data supporting the conclusions of this article will be made available by the authors, without undue reservation.
