# Supplementary material for: The first pediatric emergency and critical care fellowship in sub-Saharan Africa: investing in local leaders to build subspecialty capacity in low- and middle-income countries
Source: Front Pediatr. 2026 Jun 3;14:1847261. doi: 10.3389/fped.2026.1847261 (PMC13272386; doi:10.3389/fped.2026.1847261)
Supplement: Supplementary file 1 [file Datasheet1.pdf]

PECC fellow Program Evaluation

This anonymous REDCap survey is part of a research study about the FPECC program and will be distributed twice per year. The results of this survey will inform necessary changes to the FPECC curriculum and program implementation. Your participation is voluntary, and completing and submitting the survey indicates your consent to participate. No identifiers will be collected; please do not include names or other identifying information in your responses. Questions related to this survey may be directed to Amelie von Saint Andre-von Arnim at ameliev@uw.edu. Please provide honest, constructive feedback and use the comment sections to explain your responses. Thank you very much for your participation.

This program is well organized to permit adequate fellow training and education in Pediatric Emergency and Critical Care (PECC).

- ◯ Strongly Agree
- ◯ Agree
- ◯ Uncertain
- ◯ Disagree
- ◯ Strongly Disagree
- ◯ N/A

Comments (please explain response above): ______________________________

Fellows provide and learn clinical care for patients with an adequate range of paediatric emergency and critical care problems.

- ◯ Strongly Agree
- ◯ Agree
- ◯ Uncertain
- ◯ Disagree
- ◯ Strongly Disagree
- ◯ N/A

Comments (please explain response above): ______________________________

Fellows receive adequate supervision in their paediatric emergency and critical patient care.

- ◯ Strongly Agree
- ◯ Agree
- ◯ Uncertain
- ◯ Disagree
- ◯ Strongly Disagree
- ◯ N/A

Comments (please explain response above): ______________________________

There are adequate and sufficient resources (space, staff, equipment, etc.) to support the educational requirements of the training program.

- ◯ Strongly Agree
- ◯ Agree
- ◯ Uncertain
- ◯ Disagree
- ◯ Strongly Disagree
- ◯ N/A

Comments (please explain response above): ______________________________

Fellow workload allows for appropriate balance between education and patient care.

- ◯ Strongly Agree
- ◯ Agree
- ◯ Uncertain
- ◯ Disagree
- ◯ Strongly Disagree
- ◯ N/A

Comments (please explain response above):

____

The rapport between fellows and faculty is good.

- ◯ Strongly Agree
- ◯ Agree
- ◯ Uncertain
- ◯ Disagree
- ◯ Strongly Disagree
- ◯ N/A

Comments (please explain response above):

____

In addition to clinical care, I am learning about improving health care systems and managing a team.

- ◯ Strongly Agree
- ◯ Agree
- ◯ Uncertain
- ◯ Disagree
- ◯ Strongly Disagree
- ◯ N/A

Comments (please explain response above):

____

I receive adequate feedback from FPECC faculty on my clinical PECC skills, knowledge and overall performance as a PECC fellow.

- ◯ Strongly Agree
- ◯ Agree
- ◯ Uncertain
- ◯ Disagree
- ◯ Strongly Disagree
- ◯ N/A

Comments (please explain response above):

____
